# Target tailoring and proton beam therapy to reduce small bowel dose in cervical cancer radiotherapy: A comparison of benefits

**DOI:** 10.1007/s00066-017-1224-8

**Published:** 2017-11-03

**Authors:** Peter de Boer, Agustinus J. A. J. van de Schoot, Henrike Westerveld, Mark Smit, Marrije R. Buist, Arjan Bel, Coen R. N. Rasch, Lukas J. A. Stalpers

**Affiliations:** 1 Department of Radiation Oncology, Academic Medical Center, University of Amsterdam, Meibergdreef 9, 1105 AZ Amsterdam, The Netherlands; 2 Department of Radiation Oncology, The Netherlands Cancer Institute—Antoni van Leeuwenhoek, Plesmanlaan 121, 1066 CX Amsterdam, The Netherlands; 3 Department of Gynaecology and Obstetrics, Academic Medical Center, University of Amsterdam, Meibergdreef 9, 1105 AZ Amsterdam, The Netherlands

**Keywords:** Cervical cancer, Radiotherapy, Proton therapy, Toxicity, Normal tissue complication probability, Gebärmutterhalskrebs, Radiotherapie, Protonentherapie, Toxizität, Komplikationswahrscheinlichkeit, Normalgewebe

## Abstract

**Purpose:**

The aim of the study was to investigate the potential clinical benefit from both target tailoring by excluding the tumour-free proximal part of the uterus during image-guided adaptive radiotherapy (IGART) and improved dose conformity based on intensity-modulated proton therapy (IMPT).

**Methods:**

The study included planning CTs from 11 previously treated patients with cervical cancer with a >4-cm tumour-free part of the proximal uterus on diagnostic magnetic resonance imaging (MRI). IGART and robustly optimised IMPT plans were generated for both conventional target volumes and for MRI-based target tailoring (where the non-invaded proximal part of the uterus was excluded), yielding four treatment plans per patient. For each plan, the V_15Gy_, V_30Gy_, V_45Gy_ and D_mean_ for bladder, sigmoid, rectum and bowel bag were compared, and the normal tissue complication probability (NTCP) for ≥grade 2 acute small bowel toxicity was calculated.

**Results:**

Both IMPT and MRI-based target tailoring resulted in significant reductions in V_15Gy_, V_30Gy_, V_45Gy_ and D_mean_ for bladder and small bowel. IMPT reduced the NTCP for small bowel toxicity from 25% to 18%; this was further reduced to 9% when combined with MRI-based target tailoring. In four of the 11 patients (36%), NTCP reductions of >10% were estimated by IMPT, and in six of the 11 patients (55%) when combined with MRI-based target tailoring. This >10% NTCP reduction was expected if the V_45Gy_ for bowel bag was >275 cm^3^ and >200 cm^3^, respectively, during standard IGART alone.

**Conclusions:**

In patients with cervical cancer, both proton therapy and MRI-based target tailoring lead to a significant reduction in the dose to surrounding organs at risk and small bowel toxicity.

**Electronic supplementary material:**

The online version of this article (10.1007/s00066-017-1224-8) contains supplementary material, which is available to authorized users.

## Zusammenfassung

**Zielsetzung:**

In der vorliegenden Studie wurden die möglichen klinischen Vorteile einer Zielvolumenpräzisierung durch Ausschluss des tumorfreien proximalen Gebärmutteranteils bei der „image-guided adaptive radiotherapy“ (IGART) und einer verbesserten Dosiskonformität durch die intensitätsmodulierte Protonentherapie (IMPT) untersucht.

**Methoden:**

Die Studie umfasste Planungscomputertomographien von 11 zuvor behandelten Zervixkarzinompatientinnen, die in der Magnetresonanztomographie (MRT) einen >4 cm großen tumorfreien proximalen Uterusanteil aufwiesen. Es wurden IGART- und robust optimierte IMPT-Pläne für herkömmliche Zielvolumina mit Erfassung des gesamten Uterus sowie MRT-basierte Zielvolumina unter Ausschluss des tumorfreien proximalen Uterusanteils erstellt. Daraus ergaben sich pro Patientin 4 Behandlungspläne. Für jeden Plan wurden V_15Gy_, V_30Gy_, V_45Gy_ und D_mean_ für Harnblase, Sigmoid, Rektum und das Dünndarmkompartiment verglichen und die Komplikationswahrscheinlichkeit des Normalgewebes (NTCP) für Dünndarmakuttoxizitäten ≥Grad 2 berechnet.

**Ergebnisse:**

Sowohl die IMPT als auch die MRT-basierte Zielvolumenpräzisierung führte zu einer signifikanten Reduktion von V_15Gy_, V_30Gy_, V_45Gy_ und D_mean_ für Harnblase und Dünndarm. Die IMPT reduzierte die NTCP für Dünndarmtoxizität (≥Grad 2) von 25 auf 18 %. Diese ließ sich durch eine MRT-basierte Zielvolumenreduktion weiter auf 9 % vermindern. Eine NTCP-Reduktion von >10 % wurde durch IMPT bei 4 von 11 Patientinnen (36 %) und durch die Kombination von IMPT und MRT-basierter Zielvolumenpräzisierung bei 6 von 11 Patientinnen (55 %) erreicht. Diese mehr als 10 %ige NTCP-Reduktion war zu erzielen, wenn die V_45Gy_ für das Darmkompartiment bei der Standardzielvolumendefinition >275 cm^3^ und bei der MRT-basierten Zielvolumendefinition >200 cm^3^ betrug.

**Schlussfolgerung:**

Bei Zervixkarzinompatientinnen führen sowohl die IMPT als auch MRT-basierte Zielvolumenpräzisierungen unter Ausschluss der tumorfreien Uterusanteile zu einer signifikanten Dosisreduktion bei umliegenden Risikoorganen und im NTCP-Modell zu einer Verminderung der Dünndarmtoxizität.

## Background

External beam radiation therapy (EBRT) with simultaneous platin-based chemotherapy (CRT) and brachytherapy is the basis for successful treatment of advanced cervical cancer. Adequate coverage of the high-risk clinical target volume (CTV) in the brachytherapy boost results in high local control [1, 2]. However, the main drawbacks of radiotherapy include: (i) acute toxicity (e. g. radiation enteritis, proctitis and cystitis) and (ii) late bowel, bladder, vaginal and sexual morbidities [3].

Despite highly conformal techniques, such as intensity-modulated radiation therapy (IMRT) or volumetric-modulated arc therapy (VMAT) combined with an adaptive treatment strategy, large volumes of normal tissue are irradiated during EBRT. Typically, these volumes encompass the primary tumour, the uterus, internal and external iliac lymph node regions, the parametrium and the (proximal part of the) vagina, expanded with appropriate safety margins to compensate for microscopic spread and bladder/small bowel movements [4–6].

Therefore, the present study compares two approaches that reduce radiation burden to the organs at risk (OARs), namely:

1. Reducing the CTV and planning target volume (PTV) by excluding the part of the uterus that is not invaded by tumour as visualised on MRI (‘target tailoring’)
2. Increasing dose conformity around the PTV by means of proton beam radiotherapy

### Target tailoring

According to current guidelines, the EBRT target volume includes the whole uterine body plus safety margins to compensate for (large) inter-fraction positioning uncertainty, which substantially overlaps with the vulnerable small bowel. Inclusion of the entire uterus in the CTV is pre-eminently indicated for patients in whom the uterine body is extensively invaded by tumour. However, since the size/extension of the tumour can be increasingly better visualised by MRI [7, 8], the question arises as to whether the uninvaded part of the uterine body needs to be included during EBRT in patients with tumours limited to the uterine cervix, and in whom the potential microscopic spread in the uterine cavity can equally well be covered by brachytherapy.

### Proton therapy

Robust proton therapy treatment planning also allows a further reduction in the dose to OARs by improving dose conformity, thanks to the characteristic Bragg peak and steep dose fall-offs around the target volume [8].

This exploratory study aims to quantify the potential dosimetric advantages and the potential benefit in terms of reduction in normal tissue complication probability (NTCP) for small bowel of target tailoring and/or proton beam therapy, compared to the current standard image-guided high-precision photon beam EBRT.

## Materials and methods

### Patients

All the women in this study (*n* = 11) had previously undergone MRI for tumour staging and had been treated with curative EBRT with concurrent chemotherapy for locally advanced cervical cancer (FIGO stage IB2, IIA2-IVA and/or patients with N1 without distant metastasis) between January 2014 and December 2015. In line with all gynaecological radiotherapy centres in the Netherlands, patients received weekly cisplatin (40 mg/m^2^) as concurrent chemotherapy [9, 10]. According to international recommendations and guidelines, all patients underwent clinical examination for tumour spread assessment [11–13]. The prescribed EBRT dose was 46 Gy in 2‑Gy fractions, five fractions/week, followed by brachytherapy, usually by a Fletcher applicator with parametrial needles, 24-Gy pulse dose rate in 24 fractions (1 fraction/h). Dose prescription and dose constraints conformed to the GEC-ESTRO recommendations, aiming a minimal total dose to the high risk CTV of 85 Gy EQD2_10_ while restricting the dose to the D_2cc_ of rectum, sigmoid and bladder under 75, 75 and 90 Gy EQD2_3_ [14]. The overall treatment time was within 6 weeks in all patients.

For inclusion, all patients were required to show a substantial (>4 cm) tumour-free part of the uterus towards the fundus on pre-treatment MRI. Furthermore, patients were excluded if they had pathological lymph nodes in the common iliac or paraaortic lymph node regions, since this would dramatically enlarge the target volume and bias comparability of patients. As part of the clinical protocol, anatomical T2-weighted MRI was acquired (without contrast) using either a 1.5 T MRI system (Siemens Avanto, Erlangen, Germany) or a 3.0-T MRI system (Philips Ingenia, Best, the Netherlands). All patients with locally advanced cervical cancer who received curative CRT also underwent fludeoxyglucose (FDG) positron emitting tomography (PET-) computed tomography (CT) imaging to exclude the presence of distant metastasis (Philips Gemini, Eindhoven, the Netherlands). PET-CT imaging was performed with a full bladder in a treatment position.

### Structure definition

For each patient, the gross tumour volume (GTV) was determined by re-evaluation of the clinical examination and delineated on the pre-EBRT-acquired CT images for treatment planning, after consensus between two experienced radiation oncologists. Delineations were aided by fused PET and co-registered T2-weighted MRI. Based on the delineated GTV, target volumes were defined using two different strategies.

**Fig. 1:**
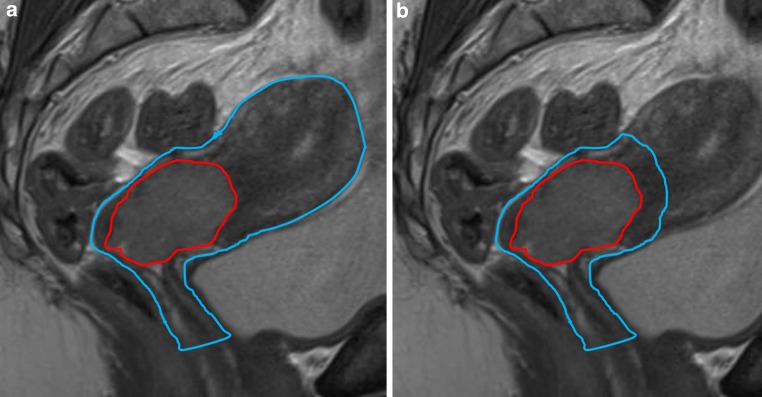
Sagittal view of T2-weighted magnetic resonance imaging with examples of the defined volumes according to the conventional definition strategy (**a**) and the novel definition strategy (**b**). In the conventional strategy (**a**), in addition to the GTV (*red*), the pCTV_current_ (*blue*) included the entire uterus and upper part of the vagina. According to the novel strategy, the pCTV_new_ (*blue*) excluded the uninvaded part of the uterus

1. The conventional target definition strategy (i. e. target volumes with the subscript ‘current’) recommends defining the primary clinical target volume (pCTV_current_) by including the GTV, cervix, entire uterine corpus and upper 2 cm of the vagina [15]. According to our institutional standard at the time of inclusion, the pCTV_current_ was enlarged by adding a 10-mm uniform margin to form the primary internal target volume (pITV_current_) [16]. In addition, the pelvic lymph node regions were delineated (lnCTV), including the common iliac, external iliac, internal iliac, presacral and parametrial lymph node regions. As the lnCTV was an obligatory part of both conventional and the new target tailored strategy, the internal target volume (ITV_current_) was created by combining the lnCTV and pITV_current_. The ITV_current_ was expanded with an 8‑mm isotropic margin to form the PTV_current_ (Fig. 1).
2. The new tailored target volume definition strategy (i. e. target volumes with the subscript ‘new’) was introduced to optimise the target volume in cervical cancer. Instead of including the entire uterine corpus into the target volume, a margin of 10 mm in the direction of the uterine fundus was added to the GTV delineation and combined with the upper part of the vagina and cervix to form the pCTV_new_ [7, 17]. Again, a 10-mm isotropic margin around the pCTV_new_ defined the pITV_new_ and the ITV_new_ was defined by combining the lnCTV and pITV_new_. The PTV was formed by expanding the ITV_new_ with an 8‑mm isotropic margin.

For proton beam therapy planning, we applied a robust treatment planning strategy, incorporating the expected uncertainties directly to ITV_current_ and ITV_new_, rather than using a ITV-PTV margin.

On all CTs, rectum, bladder, sigmoid and the bowel bag as a surrogate for small bowel were delineated according to the Radiation Therapy Oncology Group guidelines [18]. However, as the upper border is not strictly defined in this guideline, we chose to delineate the upper border at least 1 cm above the PTV.

### Treatment planning

Radiotherapy plans for both target definition strategies were generated using photons (Oncentra version 4.5, Elekta AB, Stockholm, Sweden) and protons (RayStation version 4, RaySearch Laboratories AB, Stockholm, Sweden) for all patients. All treatment plans were created based on a prescribed target dose of 46 Gy (23 × 2 Gy) and were optimised on a 3-mm uniform dose grid using the planning CT (with full bladder). Both photon and proton plan optimisations were started with the clinically used planning objectives (Supplementary Table A1) and objective values were individually optimised to minimise the dose to the OARs, while maintaining the target coverage according to the International Commission on Radiation Units & Measurements (ICRU) (D_98%_ ≥ 95%, D_max_ ≤ 107%) [19].

Photon beam treatment planning was based on a dual-arc VMAT (356° per arc, fixed 20° collimator angle) treatment technique. The optimisation process was aimed at planning the prescribed PTV dose of 46 Gy using 10 MV energy with the isocentre set to the PTV centre of mass.

Intensity-modulated proton therapy (IMPT) plans were generated based on pencil beam scanning (spot size in air: σ = 2.5–7.0 mm [226.7–70.0 MeV]) using four fixed beams (30°, 90°, 270°, 330° [prone]; 90°, 150°, 210°, 270° [supine]; [20]). As mentioned above, in proton therapy, the ITV rather than the PTV was used for robust optimisation, which included range and position errors; in addition, all robustly optimised plans were evaluated on robustness.

Assuming a proton relative biological effectiveness of 11 [21], plans were generated with a prescribed ITV dose of 46 Gy equivalent. During robust optimisation, a total of 21 scenarios were included. Besides the nominal isocentre position and the six isocentre position shifts of 8 mm in the main directions, three range errors (–3%, 0%, 3%) were also included. After optimisation, target coverage robustness was evaluated by recalculating dose distributions using 28 error scenarios, consisting of two range errors (–3%, 3%) and 14 position errors of 8 mm. The position errors were simulated by isocentre position shifts in the six main directions and the eight diagonal directions of each octant in three-dimensional space [20]. Details on the robust optimisation used have been reported by van de Schoot et al. [20].

### Analysis and statistics

First, target volumes for each patient were calculated for both the conventional and the target tailored strategy; the effect of MRI-based target tailoring was determined in terms of target volume reductions.

Secondly, plan quality was verified by quantifying the conformity index (CI) and target coverage. The CI was defined as the volume of the body receiving 95% of the prescribed dose (body V_95%_) divided by the V_95%_ of the target volume. The PTV was used to calculate the CI for photon plans, whereas the ITV was used to calculate the CI for proton plans. The maximum dose received by at least 98% of the volume (D_98%_) determined the target coverage and was reported to support the CI [22].

Differences in dose distributions corresponding to the generated treatment plans were calculated by evaluating dose-volume histogram (DVH) parameters for bladder, rectum, bowel bag and sigmoid. Besides the mean dose (D_mean_) and maximum dose (D_max_), planned dose parameters for the volumes receiving 15 Gy (V_15Gy_), 30 Gy (V_30Gy_) and 45 Gy (V_45Gy_) were extracted as derivatives for volumes receiving a low, intermediate and high dose, respectively. Patient-specific DVH differences with respect to the conventional definition strategy combined with photon therapy were tested pairwise for significance using a non-parametric statistical test (Wilcoxon signed-rank test).

### Toxicity

Since NTCP models for bladder, sigmoid and rectum are only defined for dose levels well above the prescribed dose of 46 Gy, late toxicity probabilities cannot be determined for these OARs. For small bowel, only acute toxicity models are available. Small bowel NTCP values associated with (at least) grade 2 acute small bowel toxicity were quantified using

$$\text{NTCP}=\frac{1}{1+\left (\frac{V_{50}}{V_{45Gy}}\right )^{k}}$$

where V_45Gy_ represents the volume (cm^3^) receiving 45 Gy, V_50_ = 410 cm^3^ and k = 3.2 [23]. Improvements in NTCP between the use of photon therapy and proton therapy, and conventional ‘whole uterus’ and the tailored target were calculated for each patient and correlated to the V_45Gy_ of the bowel bag. According to a model-based approach, patient-specific NTCP differences were compared with the suggested 10% NTCP difference for individual patient selection to identify those expected to benefit from proton therapy [24].

## Results

Patient characteristics, including information on MRI acquisition and tumour extensions, are presented in Table 1. Target tailoring by exclusion of the uninvaded uterine corpus resulted in an average reduction in pITV and PTV of 37% (range 17–56%) and 8% (range 3–17%), respectively.

**Table 1:** Characteristics of the 11 study patients

Patient ID No.	Age (years)	FIGO stage	Craniocaudal tumour extension (mm)	Uterine tumour-free distance^c^ (mm)	Treatment position
1^b^	34	IA2 (N1)^d^	0	55	Prone
2^a^	38	IIA2 (N1)^d^	15	51	Supine
3^b^	54	IIIB	32	42	Prone
4^b^	28	IB2	20	64	Prone
5^a^	47	IIB	43	59	Prone
6^b^	49	IIIB	62	40	Supine
7^b^	53	IIIB	27	45	Supine
8^b^	36	IIB	35	46	Supine
9^b^	41	IB2	28	44	Supine
10^b^	39	IIA2	39	58	Supine
11^a^	42	IB1^e^	35	89	Supine

*FIGO* International Federation of Gynecology and Obstetrics.

^a^MRI acquired using a 1.5 T MRI system (Siemens Avanto, Erlangen, Germany).

^b^MRI acquired using a 3.0 T MRI system (Philips Ingenia, Best, the Netherlands).

^c^Uterine tumour-free distance is defined as the distance of tumour-free uterine tissue cranial from the tumour.

^d^Both patients with N1 disease underwent lymph node debulking.

^e^This patient had two suspected nodes on PET-CT which turned out to be negative after debulking at histopathology. Therefore, she did not receive chemotherapy.

Photon-based VMAT plans were consistently planned, showing a mean CI of 1.14 (range 1.11–1.17) and a mean target coverage of 44.2 (range 44.0–44.5) Gy. Evaluation of robustness for robustly optimised IMPT plans resulted in adequate ITV coverage (D_98%_ ≥ 98%; D_max_ ≤ 107%) for all evaluated dose distributions. Further, nominal IMPT dose distributions showed consistency in both CI and target coverage, indicated by average values of 1.6 (range 1.5–1.8) and 45.7 (range 45.5–45.7) Gy, respectively. Supplementary Fig. A2 shows an example of dose distributions according to the different strategies.

Significant reductions in V_15Gy_, V_30Gy_, V_45Gy_ and D_mean_ for bowel bag were found after applying either one of both strategies: MRI-based target tailoring or using IMPT instead of VMAT (Table 2; Supplementary Fig. A3). Compared to target tailoring, the IMPT strategy lead to more significant reductions in DVH parameters of bladder, rectum and sigmoid. Combining both strategies resulted in significant further reductions of V_30Gy_, V_45Gy_ and D_mean_ for bowel bag, bladder and sigmoid (Table 2).

**Table 2:** Comparison of the mean (standard deviation) dosimetric parameters of all patients for the dose distributions corresponding to the specific target volume and treatment modality

	Photon therapy		Proton therapy	
	pCTV_current_	pCTV_new_	pCTV_current_	pCTV_new_
Bladder				
V_15Gy_ (%)	95.3 (7.8)	88.4 (10.3)*	86.8 (7.7)*	81.3 (15.2)*
V_30Gy_ (%)	74.0 (8.4)	64.5 (10.5)*	62.0 (10.7)*	50.8 (16.0)*†
V_45Gy_ (%)	35.8 (7.2)	27.6 (6.8)*	30.2 (11.3)	21.9 (13.8)†
D_mean_ (Gy)	37.2 (2.6)	34.0 (3.4)*	33.0 (3.4)*	29.3 (5.8)*†
D_max_ (Gy)	47.1 (0.6)	47.1 (0.6)	48.1 (0.9)*	48.0 (0.9)*
Rectum				
V_15Gy_ (%)	100.0 (0.0)	100.0 (0.0)	99.7 (1.0)	99.8 (0.6)
V_30Gy_ (%)	99.7 (0.3)	99.6 (0.6)	84.3 (5.9)*	81.5 (7.5)*†
V_45Gy_ (%)	47.1 (19.2)	51.7 (16.6)	40.9 (5.9)	40.3 (5.7)
D_mean_ (Gy)	43.5 (0.9)	43.5 (0.9)	39.7 (1.4)*	39.2 (1.5)*†
D_max_ (Gy)	46.0 (0.3)	46.1 (0.4)	46.4 (0.2)*	46.5 (0.2)*
Sigmoid				
V_15Gy_ (%)	96.0 (6.1)	92.0 (16.3)	83.7 (27.0)*	82.6 (27.2)*
V_30Gy_ (%)	81.1 (27.1)	78.6 (28.3)	71.6 (25.3)*	68.1 (26.3)*†
V_45Gy_ (%)	55.9 (21.7)	50.1 (23.2)*	44.3 (18.2)*	33.3 (15.6)*†
D_mean_ (Gy)	39.6 (6.8)	38.2 (8.4)*	34.7 (10.7)*	33.2 (10.9)*†
D_max_ (Gy)	46.9 (0.5)	46.9 (0.6)	46.9 (0.7)	46.8 (0.9)
Bowel bag				
V_15Gy_ (cm^3^)	899.1 (287.3)	838.6 (320.7)*	559.4 (207.0)*	530.9 (212.1)*
V_30Gy_ (cm^3^)	501.4 (199.9)	460.3 (202.8)*	382.3 (170.0)*	337.9 (154.1)*†
V_45Gy_ (cm^3^)	268.0 (146.3)	226.3 (123.7)*	227.7 (117.4)*	173.4 (88.8)*†
D_mean_ (Gy)	20.7 (11.4)	16.9 (6.3)*	12.8 (5.8)*	11.8 (5.6)*†
D_max_ (Gy)	47.8 (0.5)	47.9 (0.5)	47.9 (0.4)	47.7 (0.4)

Significant improvements (*p* < 0.05) compared to current clinical practice (i. e. photon therapy, pCTV_current_) and proton therapy using conventional target volumes are indicated by an asterisk (*) and a dagger (†), respectively

*pCTV* primary clinical target volume

**Fig. 2:**
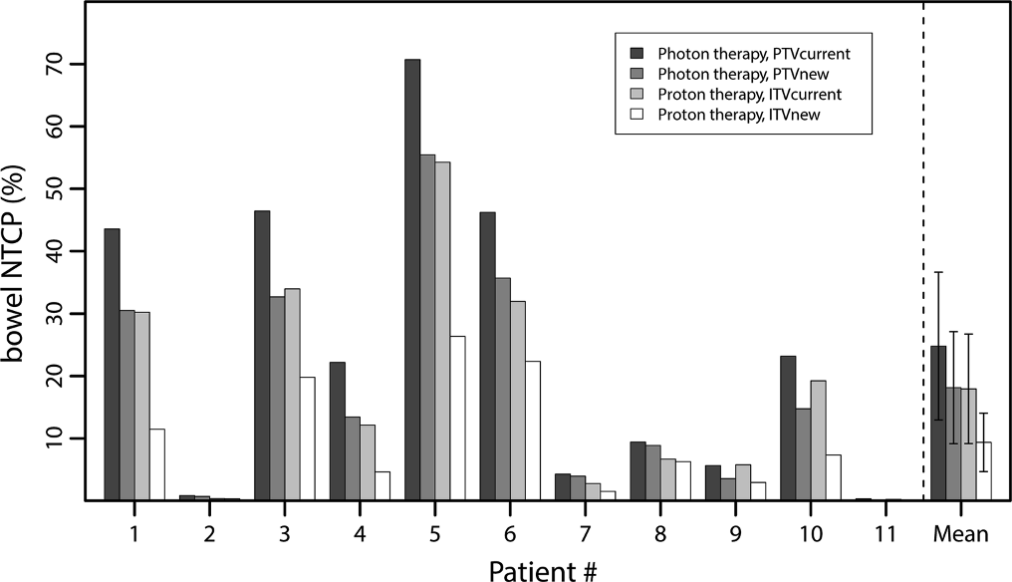
Bar plots of the small bowel normal tissue complication probability (*NTCP*) values are shown per patient according to different target volume definition strategies and different treatment modalities. The grouped bars at the right side represent mean NTCP values and the error bars indicate one standard deviation

Fig. 2 shows patient-specific NTCP values associated with small bowel acute toxicity for both the conventional and tailored target volumes, and for both photon therapy and proton therapy. VMAT without target tailoring (PTV_current_) resulted in an average grade ≥2 acute small bowel NTCP of 25% (Table 3). Both target tailoring and proton therapy reduced the NTCP averagely to 18%, while combining both strategies resulted in an average NTCP for acute small bowel toxicity of 9% (Table 3).

**Table 3:** Comparison of the mean (range) bowel normal tissue complication probability (NTCP) values (%) for the planned dose distributions of all patients, including the absolute NTCP differences

	pCTV_current_	pCTV_new_	*Absolute difference (%)*
Photon therapy	25.0 (1.0–71.0)	18.0 (1.0–55.0)	*7.0*
Proton therapy	18.0 (1.0–54.0)	9.0 (1.0–26.0)	*9.0*
*Absolute difference (%)*	*7.0*	*9.0*	–

*pCTV* primary clinical target volume

**Fig. 3:**
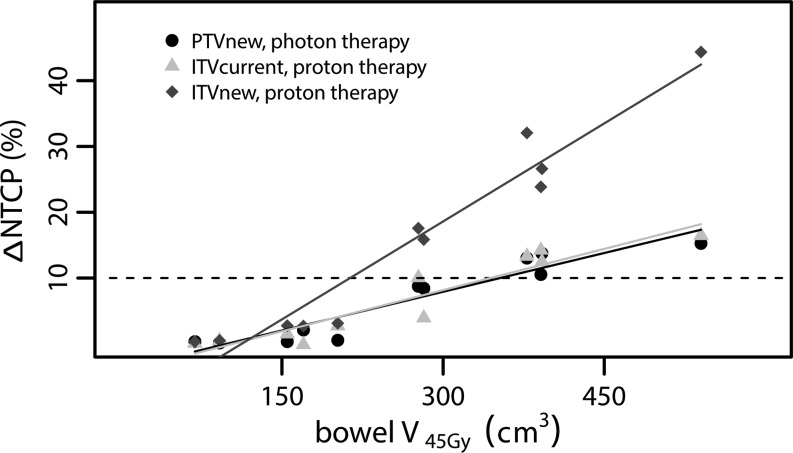
Absolute improvements in normal tissue complication probability (*∆ NTCP*) compared to conventional high precision photon therapy without target volume reduction (PTV_current_) as a function of bowel bag volume receiving 45 Gy in current clinical practice. Each *dot* represents a measurement for an individual patient and linear fits are added for visualisation purposes. The *dotted horizontal line* indicates the 10% ∆ NTCP threshold above which proton therapy is indicated [18]

Improvements in small bowel NTCP were particularly observed in patients with a high NTCP for standard treatment (Fig. 2); this can be explained by a consequently large volume of the bowel bag receiving at least 45 Gy in current radiotherapy practice (Fig. 3). The proposed 10% NTCP reduction threshold as an acceptable indication for proton therapy was observed in 4/11 patients when using conventional target volumes. For these patients, the V_45Gy_ of the bowel bag was at least 275 cm^3^ in the standard treatment. If, additionally, the target was tailored by excluding the non-invaded uterine corpus, the 10% NTCP reduction threshold was passed in 6/11 patients of whom the V_45Gy_ of the bowel bag was at least 200 cm^3^.

## Discussion

In our study, it was estimated that both target tailoring by excluding the non-invaded uterine corpus and proton therapy would yield a significant and clinically relevant reduction in dose to the small bowel. Compared to the clinical standard, both approaches separately yielded an absolute NTCP reduction for ≥2 acute small bowel toxicity of 7%, and a reduction of 16% when combined. The model-based approach suggested a 10% NTCP reduction threshold as an acceptable indication for taking proton therapy into consideration [24]. We estimated a NTCP reduction of ≥10% by proton therapy for 4/11 (36%) patients in whom the initial bowel bag V_45Gy_ was ≥275 cm^3^, and in 6/11(55%) patients ≥200 cm^3^ when MRI-based target volumes were applied. However, both strategies aimed at toxicity reduction require further investigation.

### Reduced EBRT target volume definition

With regard to the safety of target volume reduction there is indirect supportive evidence. Clinical examination does not always provide clear answers on tumour spread in the direction of the uterine fundus due to the its deep location in the pelvis. In these cases additional imaging can be useful. For instance, with modern MRI techniques, uterine invasion can be visualised prior to treatment and is widely used for, e. g. brachytherapy planning [7, 25]. Furthermore, studies validating MRI-based tumour volume delineations with histopathology in cervical cancer have demonstrated the feasibility of accurate tumour definition [7, 17].

Further evidence can be found in trachelectomy series for early stage cervical cancer, where patients with tumours ≥2 cm show a higher risk of recurrence. However, these tumours typically recurred regionally and not in the remaining uterine corpus [26, 27]. Recent studies also indicate that histopathological characteristics, such as lymph vascular space invasion (HR 3.2, *p* = 0.03) and deep stromal invasion (HR 4.5, *p* = 0.005) are the most important independent predictive factors for recurrence after surgery [28, 29].

Furthermore, with modern image-guided adaptive radiotherapy strategies, local control rates in patients with International Federation of Gynecology and Obstetrics (FIGO) stage IIB and IIIB are 96% and 86%, respectively, particularly if the D_90%_ of the high-risk CTV is adequately covered by brachytherapy [1, 30]. We should not compromise these favourable results by reducing the elective dose in the macroscopically uninvolved uterus without due consideration, nor by omitting the clinical examination, which naturally plays a pivotal role. However, delivering such an elective dose with brachytherapy (instead of EBRT) to the uninvaded surface of the uterine cavity would likely lower the collateral dose to small bowel. Therefore, this strategy deserves further investigation.

### Proton therapy

Earlier comparisons between photon therapy and proton therapy according to an adaptive strategy in cervical cancer show similar reductions in V_15Gy_, V_30Gy_ and V_45Gy_ for bladder, rectum and bowel bag, as well as in acute small bowel NTCP (7%) in favour of proton therapy [31, 32].

Van de Schoot et al. showed the feasibility of accurate dose delivery using an adaptive strategy under image guidance while maintaining desirable dose distributions [31]. Therefore, this adaptive strategy was also performed in this study in order to compensate for anatomical deformations. Furthermore, proton plans were anticipated on day-to-day changes by robust optimisation and subsequent evaluation of robustness, according to recent literature [6, 20].

### Toxicity estimation

According to the volume constraint defined by Roeske et al., ≤195 cm^3^ of the bowel bag should receive 45–50 Gy [20]. In the present study, this constraint was reached in only 4/11 patients (36%) when using IMPT or target tailoring.

The impression was gained that patients with an extensive small bowel volume receiving 45 Gy were mostly women with anteversion of the uterus, i. e. bending away from the iliac lymph node region. Especially in these patients, a reduction in V_45Gy_ decreased the probability for acute small bowel toxicity. This might also result in a lower risk of *late* small bowel toxicity, as acute small bowel toxicity is a known risk factor for this endpoint [33]. That is to say that a reduction in the small bowel V_45Gy_ could improve the quality of life of these women. In addition, quality of life could also be improved by reducing dose to other OAR which depend primarily on D_2cc_ of the delivery of high dose brachytherapy boost [34–36].

## Conclusion

Compared to standard radiotherapy for locally advanced cervical cancer, both proton therapy and target tailoring by excluding the non-involved uterine corpus lead to a significant reduction in the dose to surrounding OARs, which probably yields a significant reduction in small bowel acute toxicity. Moreover, the combination of both strategies resulted in an additional reduction in estimated acute small bowel toxicity. For example, it was estimated that the combination of target tailoring and proton therapy may lead to an NTCP reduction of at least 10% in patients with a bowel bag V_45Gy_ above 200 cm^3^. Nevertheless, the safety and efficacy of these novel approaches need further investigation before they can be tested in clinical studies.

## Caption Electronic Supplementary Material

**Supplementary Table A1** Planning objectives for photon (proton) therapy planning

**Supplementary Fig. A2 **Sagittal view of colourwash map examples of dose distributions are shown for the use of PTV_current_ (a) and PTV_new_ (b) combined with photon therapy, and for the use of ITV_current_ (c) and ITV_new_ (d) combined with proton therapy. All dose distributions indicated adequate target (*white contour*) coverage while differences in dose to surrounding healthy tissue are observed (*PTV* planning target volume, *ITV* internal target volume)

**Supplementary Fig. A3** Boxplots of dose-volume histogram parameters over all planned dose distributions of all patients are shown for bladder, rectum, sigmoid and bowel bag. *Boxes* represent upper and lower quartiles (IQR), the band inside the box is the median value and the whiskers are the highest (lowest) value within 1.5 IQR of the upper (lower) quartile (*PTV* planning target volume, *ITV* internal target volume)
