# Supplementary material for: Target tailoring and proton beam therapy to reduce small bowel dose in cervical cancer radiotherapy: A comparison of benefits
Source: Strahlenther Onkol. 2017 Nov 3;194(3):255–63. doi: 10.1007/s00066-017-1224-8 (PMC5847034; doi:10.1007/s00066-017-1224-8)
Supplement: Supplementary file 1 — Supplementary Table A1 Planning objectives for photon (proton) therapy planning [file 66_2017_1224_MOESM1_ESM.doc]

**Supplementary Table A1** Planning objectives for photon (proton) therapy planning.

| Planning objectives | |
| --- | --- |
| PTV (ITV) | Minimum dose 46 Gy |
|  | Maximum dose 46.8 Gy |
| Body | Dose fall-off: 46 – 30 Gy over 1.0 cm |
| Rectum | Maximum dose 43.7 Gy |
|  | Maximum 30 Gy to 70% of the volume |
| Bladder | Maximum dose 43.7 Gy |
|  | Maximum 30 Gy to 70% of the volume |
| Bowel cavity | Maximum dose 43.7 Gy |

*PTV* planning target volume; *ITV* internal target volume.
