# Supplementary figures and images for: Target tailoring and proton beam therapy to reduce small bowel dose in cervical cancer radiotherapy: A comparison of benefits
Source: Strahlenther Onkol. 2017 Nov 3;194(3):255–63. doi: 10.1007/s00066-017-1224-8 (PMC5847034; doi:10.1007/s00066-017-1224-8)

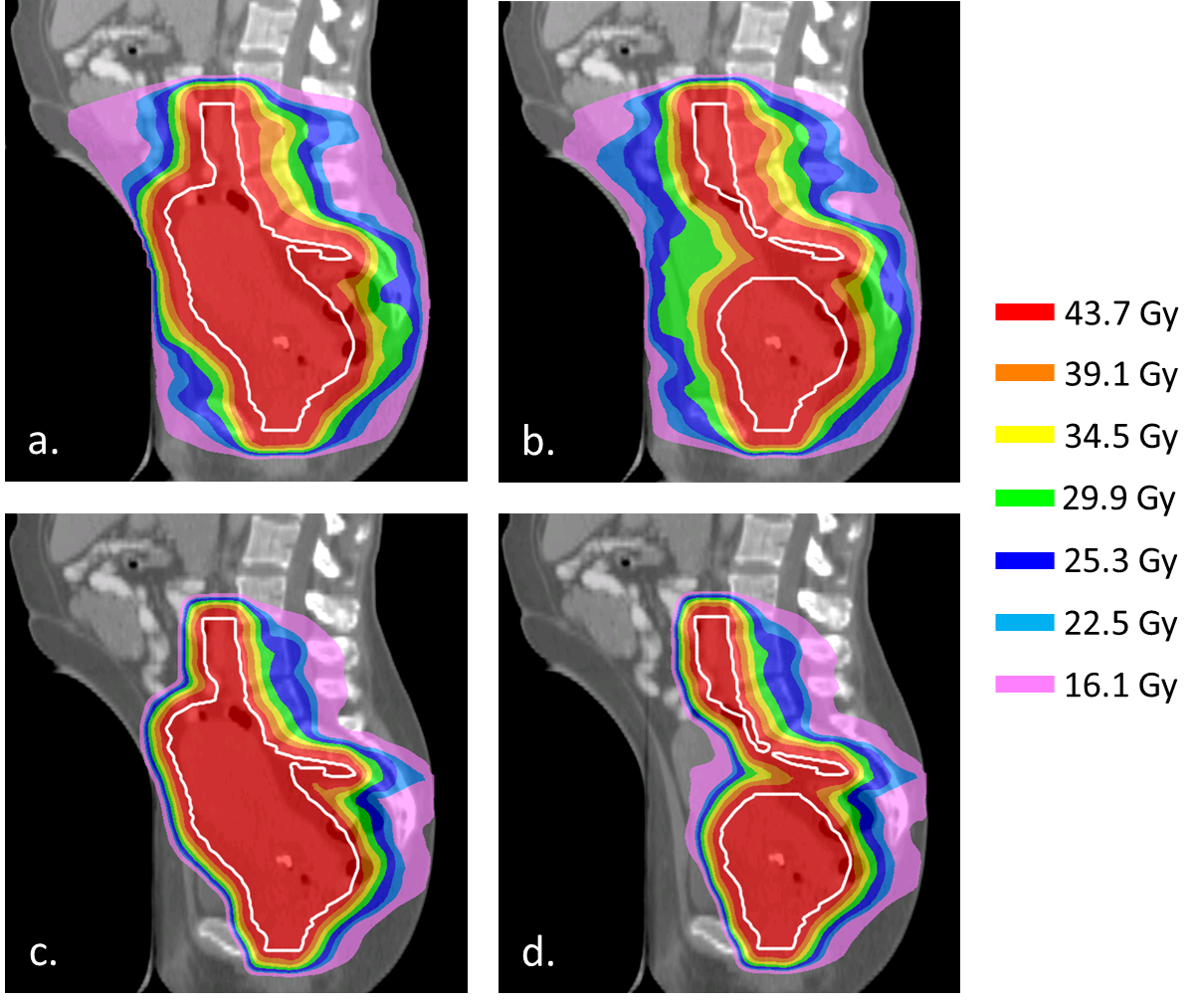

Supplement: Supplementary file 2 — Supplementary Fig. A2 Sagittal view of colourwash map examples of dose distributions are shown for the use of PTVcurrent (a) and PTVnew (b) combined with photon therapy, and for the use of ITVcurrent (c) and ITVnew (d) combined with proton therapy. All dose distributions indicated adequate target (white contour) coverage while differences in dose to surrounding healthy tissue are observed (PTV planning target volume, ITV internal target volume) [file 66_2017_1224_MOESM2_ESM.tif]

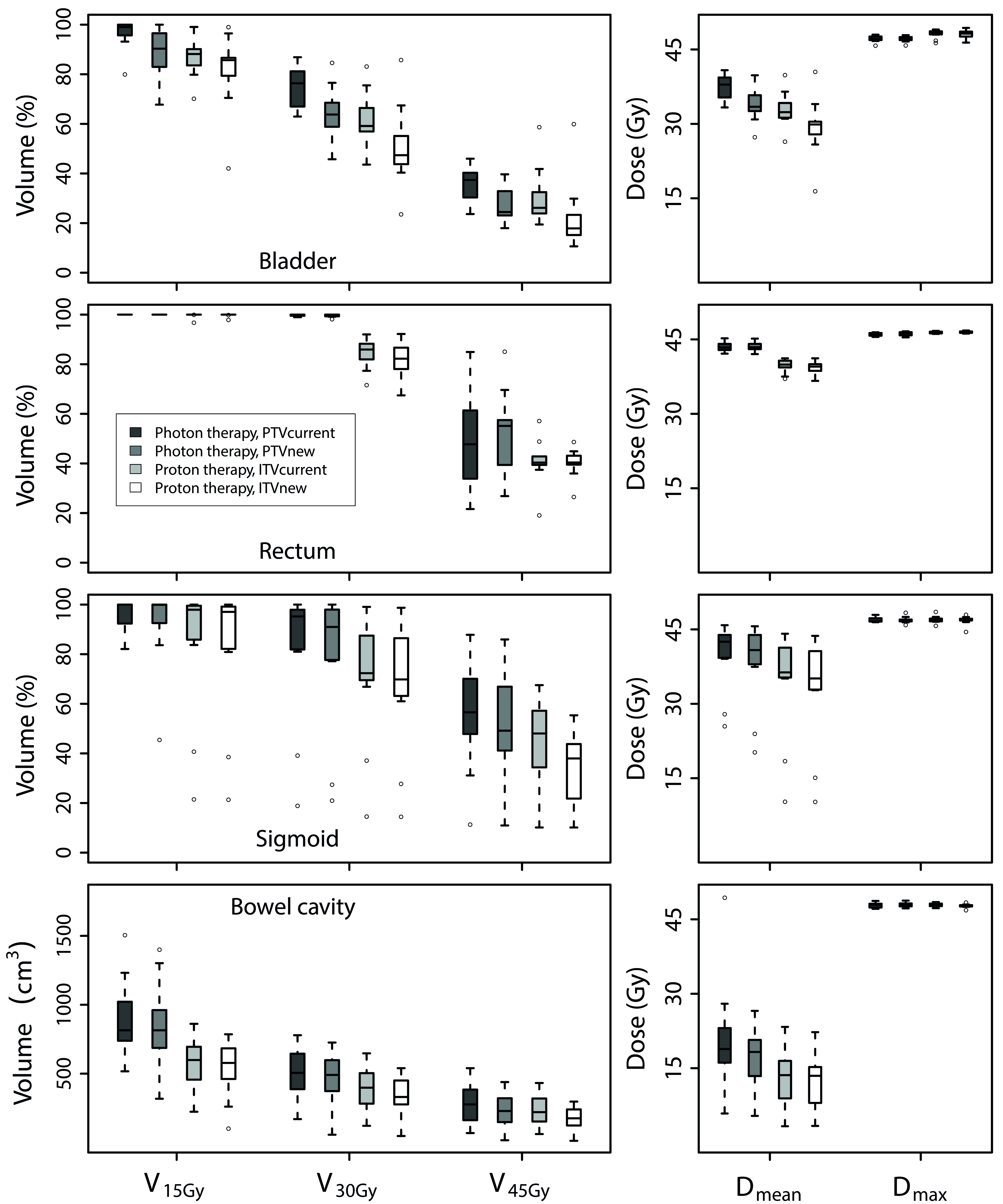

Supplement: Supplementary file 3 — Supplementary Fig. A3 Boxplots of dose-volume histogram parameters over all planned dose distributions of all patients are shown for bladder, rectum, sigmoid and bowel bag. Boxes represent upper and lower quartiles (IQR), the band inside the box is the median value and the whiskers are the highest (lowest) value within 1.5 IQR of the upper (lower) quartile (PTV planning target volume, ITV internal target volume) [file 66_2017_1224_MOESM3_ESM.tif]
